# Enabling data sharing and utilization for African population health data using OHDSI tools with an OMOP-common data model

**DOI:** 10.3389/fpubh.2023.1116682

**Published:** 2023-06-09

**Authors:** Sylvia Kiwuwa-Muyingo, Jim Todd, Tathagata Bhattacharjee, Amelia Taylor, Jay Greenfield

**Affiliations:** ^1^African Population and Health Research Center (APHRC), Nairobi, Kenya; ^2^London School of Hygiene and Tropical Medicine, University of London, London, United Kingdom; ^3^Department of Computing and Information Technology, Malawi University of Business and Applied Sciences, Blantyre, Malawi; ^4^Committee on Data of the International Science Council, Paris, France

**Keywords:** data sharing, common data models, COVID-19, pandemic response, data standards

## Abstract

The COVID-19 pandemic has spurred the use of AI and DS innovations in data collection and aggregation. Extensive data on many aspects of the COVID-19 has been collected and used to optimize public health response to the pandemic and to manage the recovery of patients in Sub-Saharan Africa. However, there is no standard mechanism for collecting, documenting and disseminating COVID-19 related data or metadata, which makes the use and reuse a challenge. INSPIRE utilizes the Observational Medical Outcomes Partnership (OMOP) as the Common Data Model (CDM) implemented in the cloud as a Platform as a Service (PaaS) for COVID-19 data. The INSPIRE PaaS for COVID-19 data leverages the cloud gateway for both individual research organizations and for data networks. Individual research institutions may choose to use the PaaS to access the FAIR data management, data analysis and data sharing capabilities which come with the OMOP CDM. Network data hubs may be interested in harmonizing data across localities using the CDM conditioned by the data ownership and data sharing agreements available under OMOP's federated model. The INSPIRE platform for evaluation of COVID-19 Harmonized data (PEACH) harmonizes data from Kenya and Malawi. Data sharing platforms must remain trusted digital spaces that protect human rights and foster citizens' participation is vital in an era where information overload from the internet exists. The channel for sharing data between localities is included in the PaaS and is based on data sharing agreements provided by the data producer. This allows the data producers to retain control over how their data are used, which can be further protected through the use of the federated CDM. Federated regional OMOP-CDM are based on the PaaS instances and analysis workbenches in INSPIRE-PEACH with harmonized analysis powered by the AI technologies in OMOP. These AI technologies can be used to discover and evaluate pathways that COVID-19 cohorts take through public health interventions and treatments. By using both the data mapping and terminology mapping, we construct ETLs that populate the data and/or metadata elements of the CDM, making the hub both a central model and a distributed model.

## Introduction

Data sharing is increasingly recognized as a requirement for advancing clinical and population health knowledge and enabling scientific research for new drugs, vaccines and health systems. Until 2015 the main model for data sharing has been to deposit more or less anonymised (meta)data in a repository with more or less access control, depending on the dataset. DataFirst for South Africa and other African countries and Harvard Dataverse are two such repositories that come to mind. INDEPTH Data Repository is important as many of the INSPIRE (health data partnership) sites were part of the iSHARE initiative (1). This model for data sharing does not specifically enable the pooling of data across studies and their datasets. Instead, repositories tend to support meta-analysis (2), which combine study results across multiple studies, but don't combine source data for re-analyses.

Pooling data across data sources like disease registries, health and demographic surveillance systems, hospital visits and labs was not feasible before the advent of big data. The challenges with pooling data including lack of standardized tools, terminologies, absence of unique identifiers, access to technology platforms, burden of competing data sharing initiatives and contextual barriers to data sharing attenuated potential impact on research (3). Big data introduced the use of common data models (CDM) as an alternative to repositories to enable data sharing and the combination of data for re-analysis. CDM were also in use in other initiatives but had their own limitations, ALPHA has the ALPHA Data Specs (Data specification for ALPHA mortality data (4) - and Data specification for ALPHA HIV incidence data (5), INDEPTH Data Repository used the INDEPTH Core Micro Dataset model – Table 1: Common event attributes for the INDEPTH data specification and Table 2: Event types for the INDEPTH data specification (6). Before the advent of big data and CDM, there is limited evidence of the impact of data sharing via repositories in improving health outcomes in LMICs (2).

Enter the COVID-19 pandemic. It came with a novel virus and a pathogen that was extremely contagious, extremely adaptable, and very successful when it comes to confusing the host's immune system. Under the circumstances it became necessary to share data across domains and across borders in a framework that lent itself to *continuous data analysis*. In the United States this led to the National COVID-19 Cohort Collaborative (N3C) (7). In Europe this led to the EHDEN Portal (8). In both instances the cornerstone of these efforts has been the Observational Medical Outcomes Partnership (OMOP) Common Data Model (CDM), allied with the data tools developed by the Observational Health Data Sciences and Informatics (OHDSI).

The African Population Health Research Center (APHRC) had become concerned about the dearth of data and evidence when it came to the health of marginalized, vulnerable and minority populations. In 2020 APHRC began the journey of migrating some of its data into the OMOP CDM. At the same time APHRC began to incorporate governance with data migration so that the adoption of OMOP would go hand-in-glove with its ethical compass. This led to INSPIRE (9).

The Implementation Network for Sharing Population Information from Research Entities (INSPIRE) is a health data partnership across research organizations and sentinel surveillance sites in eastern and southern Africa. It has used the framework from the US National COVID Cohort Collaborative (N3C) and adapted it to the local context of the SSA region by enhancing it accordingly to build capacity to share population health data from Africa using an OMOP CDM.

INSPIRE is adapting the N3C model to support federated and centralized longitudinal population health research alongside clinical research as a way of including disease prequels and sequelae. This is a new model of data sharing which seeks to address the challenges and limitations of data sharing in the context of big data. Additionally, it is growing the cohorts that are ingested into CDM to include other communicable and non-communicable diseases. This is in preparation for the next pandemic and as a lesson learned from COVID-19 that the treatment of one disease affects the treatment of others. Finally, INSPIRE is growing the platform to include place-based real-world data (RWD). This is so research on the platform can consider locality when it comes to the factors that may be in play both during surveillance, intervention and over time and post-pandemic in longitudinal research. With its ethical bent INSPIRE is making data on climate, other exposures and the built environment available on our research platform to qualify and disaggregate cohort definitions and reanalysis designs in line with the experiences of marginalized, vulnerable and minority populations.

With its extensions INSPIRE has become a use case in two global initiatives – WorldFAIR and the Global Open Science Cloud (GOSC). WorldFAIR seeks to compare and contrast best practices that advance the implementation of the FAIR data principles, in particular those for Interoperability, across many areas of science (10). GOSC seeks to “connect the different international, national and regional research Infrastructures to create a global digital environment for borderless research and innovation” (11). Across these two efforts INSPIRE is testing the OMOP research platform: is its (meta)data infrastructure (including new extensions like the one for hosting place-based RWD) enough? For example, can the platform track viral imports and exports of COVID-19 between Africa and the rest of the world, and between individual African countries in real time? Do the standard vocabularies the platform provides keep pace with advances in the development of COVID-19 vaccines and therapies? Can the OMOP OHDSI platform together with its extensions account for the availability of vaccines and therapies by location? In this context and others can the platform take into account mobility and the distance to care? Can the data capture and analysis workbench OHDSI provides account for health inequities including gender and intersectionalities that might be beneficial in contextualizing health research for vulnerable and marginalized populations? In a pandemic all these factors can figure into the target cohorts, comparison cohorts, event cohorts and/or outcome cohorts that OMOP and OHDSI are able to construct for purposes of analysis. Through this collaborative network the real goal is to re-use data for public social good while preventing harm that may result from data sharing such as the inadvertent exposure of participants and the lack of inclusion or diversity.

This paper is organized as follow. In section 1 we describe INSPIRE's innovative data approach and progress building a FAIR, borderless population health research platform. We will show how the platform can perform disease surveillance and hatch intervention strategies using real world data (RWD), including for other diseases than COVID-19 and local environmental data. In section 2 the innovations are supported by four methods ranging from (i) platform governance, (ii) capacity building as crosscutting issues, (iii) metadata pipeline practices and (iv) the integration with place-based attributes. In section 3 the four methods helped create the framework for the infographics which is a graphical representation of the materials and methods used by the INSPIRE data approach. Section 4 brings all three together to explore the issues that emerge from the use of OMOP CDM within an East and Southern Africa context. This section deliberates about on-the-ground challenges using examples for the platform for evaluation and anlysis of COVID-19 harmonsied data (PEACH) ongoing implementation in two countries. A discussion in section 5 considers a review of the literature and how our research iteratively informs the infographic.

## INSPIRE's innovative data approach and contribution to the field

Although the OMOP Common Data Model was first developed to host clinical data captured in electronic health records, more recently it has been used to standardize registry data. Biedermann et al. (12) describe their experience migrating three pulmonary hypertension registries into OMOP. Also, in 2021 Belenkaya et al. (13) describe their experience extending the OMOP CDM and its standardized vocabularies to host US Tumor Registry data. Now APHRC and the London School of Hygiene and Tropical Medicine (LSHTM) through INSPIRE are in the process of marrying the OMOP CDM with population health informatics in two use cases. In one use case INSPIRE is developing a (meta)data pipeline that migrates Integrated Disease and Surveillance Response (IDSR) person-level data that includes COVID-19 specimen data collection and lab results from multiple countries into OMOP (14). In another use case LSHTM through INSPIRE is using OMOP to host the results of HIV serosurveys conducted in the course of demographic surveillance that registers person migration in and out of many sentinel sites (HDSS) (15) in Sub-Saharan Africa over time (16).

In both of its population health use cases INSPIRE uses a federated model. It has built a Platform as a Service (PaaS) that includes both the OMOP CDM and a set of OHDSI services that it deploys locally at the sentinel sites. The data extraction, transformation and loadings (ETLs) that migrate the data in each use case are developed centrally and shared locally. The same goes for any new terminology needed in these health and demographic surveillance use cases. The same goes for cohort definitions that OHDSI hosts centrally in a phenotype library and distributes locally for use in ATLAS. ATLAS is a mostly point-and-click OHDSI component that can be used to both define and execute descriptive, predictive and prescriptive research studies based on standardized target, comparator, event and outcome cohort definitions.

So far materials shared by INSPIRE with sentinel sites includes the PaaS, ETLs and new terminology, depending on the use case. There is one more ongoing development that INSPIRE is about to adopt and share with the sites by way of its PaaS. This is a new component being developed by the OHDSI GIS Working Group (17). This component ingests, persists and outputs place-based attributes. Place-based attributes can include meteorological data by locality and, more generally, any exposure data keyed to specific localities.

INSPIRE is driving population health research, other diseases other than COVID-19, real world data (RWD) and local environmental data supported by four methods.

## The implementation methods within INSPIRE

Methods are divided into four categories: platform governance, capacity development (meta)data pipeline best practices and integration with place-based attributes.

### Addressing crosscutting issues

#### Platform governance

Platform governance conditions capacity development (meta)data pipeline best practices and integration with place-based attributes. It entails preserving privacy and confidentiality. See, for example, “the ethical challenges encountered during community tracing of HIV Positive disengaged women in Uganda communities” (18). Depending on the research being conducted, maybe only methods are shared across the INSPIRE sentinel sites [i.e., capacity development (meta)data pipeline best practices, integration with place-based attributes] and no data is pooled. To encourage data pooling across sites and the construction of hubs for continuous data analysis as needed, INSPIRE, following N3C, supports a data pooling solution that comes in three flavors with each flavor having it own OMOP CDM instance and access requirements (Table 1). In one flavor protected health information is not perturbed. In a second flavor protected health information is de-identified. The third flavor is synthetic data. It is computationally derived from the unperturbed OMOP CDM instance. It contains no unperturbed data but resembles unperturbed data statistically (19):

**Table 1:** Three flavors of data pooling N3C supports and their access requirements.

**Data level**	**Data description**	**Eligible users**	Access requirements^*^
Limited Data Set (LDS)	Patient data that retain the following protected health information: •Dates of service •Patient zip codes	• Research from U.S.-based institutions	• N3C registration • N3C Data Enclave account • Data Use Agreement (DUA) executed with NCATS • NIH IT training requirements • Approved Data Use Request (DUR) • Human Subjects Research Protection training completion • Local Human Research Protection Program IRB determination letter
De-identified Data Set	Patient data from LDS with the following changes: • Dates of service are algorithmically shifted to protect patient privacy • Patient ZIP codes are truncated to the first three digits or removed entirely if the ZIP code represents fewer than 20,0000 individuals	• Research from U.S.-based institutions • Research from foriegn institutions	• N3C registration • N3C Data Enclave account • Data Use Agreement (DUA) executed with NCATS • NIH IT training requirements • Approved Data Use Request (DUR) • Human Subjects Research Protection training completion
Synthetic Data Set	Data that are computationally derived from the LDS that resemble patient intformation statistically but are not actual patient data	• Research from U.S.-based institutions • Research from foriegn institutions • Citizen scientists	• N3C registration • N3C Data Enclave account • Data Use Agreement (DUA) executed with NCATS • NIH IT training requirements • Approved Data Use Request (DUR)

* Data acccess requirements may change over time.

Note that INSPIRE is adopting access requirements to the African context.

The three flavors provision the alternatives available to data users in the African context. During IDSR implementation our partnerships require access to researchers from within the ministries of health or institutions. Partners may play a coordination role in supporting over stretched Ministries of health and stakeholders. The implementation is inclusive of individuals in communities impacted by the COVID-19 pandemic as stakeholders. INSPIRE through OMOP implementation of the IDSR has set up instances to support the three flavors, the first one with limited access to users in country is recommended by the ministries of health. In all the use cases, it ensures accountability for the results and transparency and feedback loops. INSPIRE is creating conditions for positive impact on gender and intersectionalities and accountability mechanisms (see https://covidsouth.ai/research?lang=en).

Additionally, platform governance encompasses a commitment to hosting results that are sex-disaggregate across the (meta)data pipeline. See, for example, this discussion of “Sex-disaggregated data matters” (20).

Finally, platform governance encompasses the use of lived experience experts. See, for example, “the integration of lived experience perspectives into the research, from discovery to translation…” described here Beames et al. (21).

The citizen scientists comprise of the individuals in communities with lived experience, data managers of participating institutions or ministries of health, data scientists, statisticians, public health experts, policy and decision makers, researchers and partners.

Platform governance at INSPIRE also benefits from past experience. Many sentinel sites that participate in INSPIRE today previously participated in the INDEPTH Network (22). The governance of INDEPTH has had many challenges that INSPIRE is learning from. Perhaps the lesson learned that is shaping INSPIRE the most is the need for capacity strengthening. The capacity strengthening that comes with participation in INSPIRE by sentinel sites takes many forms but all with same end: the dissemination of new methods and technologies fit for use in the Africa context.

#### Capacity development

In a federated approach where each site gets its own PaaS, the challenges are significant. For example, each region may host its IDSR person-level data in its own data collection system. Given the heterogeneity of data sources, the INSPIRE (meta)data pipeline has two hops. In the first hop, sites migrate data from their local systems into a standard format. In the second hop just one ETL is developed centrally that moves data from the standard format into the OMOP CDM. Each site is responsible is for its own first hop. An ETL is developed centrally for the second hop and shared locally. Between the first hop and the second hop there are data quality checks that the standardized person-level IDSR has to pass. These checks gate the execution of the ETL that moves the data in the standard format into the OMOP CDM.^1^ Odysseus has developed ARACHNE Research Network, a platform for consistent, secure and compliant observational research process for federated OHDSI (23).

It is during this gate that capacity development occurs. This capacity strengthening extends to Improving the technical capacity to support the management of large data sets in both government and private sectors while promoting privacy, confidentiality, data security and the alignment with applicable country-specific data policies.

When data quality checks fail, sites get to trace why their data can't be moved into the many tables (domains) that make up the OMOP CDM. They learn the thinking the ETL uses that moves data from source to target.

Before OHDSI and INSPIRE, both APHRC and LSHTM conducted network research using this same two hop approach with the data quality checks in between. Before the pandemic, the capacity development that is needed at the gate between the two hops occurred in in-person workshops. Neither APHRC nor LSHTM has experience conducting these workshops remotely.

### Technical specifications that demand capacity development

#### (Meta)data pipeline best practices

**Figure 1:**
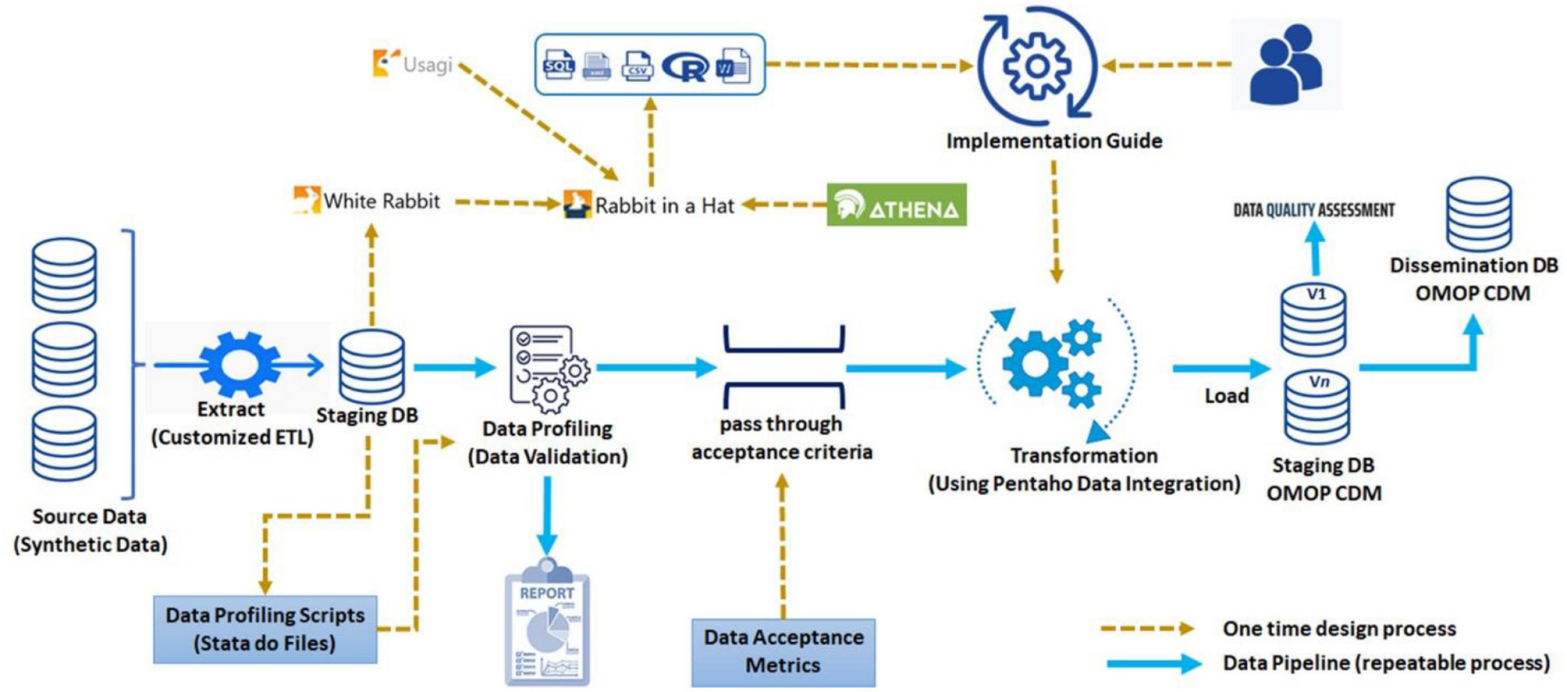
INSPIRE (meta)data pipeline.

The INSPIRE (meta)data pipeline was developed following EHDEN Academy (24) and IQVIA (25) best practices (Figure 1).

In a first hop source data is extracted, transformed and loaded into one of several data exchange formats, depending on the source data type. So far INSPIRE supports these formats:

- A data exchange format for longitudinal health and demographic surveillance datasets used across SSA first by the INDEPTH Network (26) and then the London School of Hygiene and Tropical Medicine (27) ALPHA networks. The format accommodates verbal autopsy results and is currently used in excess death research supported by the Bill and Melinda Gates Foundation (28).
- A data exchange format developed by the WHO for COVID-19 EHR data (29).
- A data exchange format developed by the WHO for person-level Integrated Disease and Surveillance Response (IDSR) datasets (30).
- A data exchange format developed by the International HundredK+ Cohorts Consortium (IHCC) with support from the Wellcome Trust for the LMIC COVID-19 Questionnaire which includes several sections for mental health (31).
- An integration approach that accounts for the physical and social environment in play with individuals by location. See the section on “Integration with place-based attributes” below. It talks to the use of sustainable develop goal (SDG) along with other indicators in longitudinal population studies.

The COVID-19 data exchange formats are supported by the Global South AI4COVID program funded by IDRC/SIDA.

The INSPIRE PaaS hosts several staging databases, one that corresponds to each of these data exchange formats. These staging databases are defined centrally and deployed locally. Each locality develops ETLs that move their source data into one or more of these staging databases.

The next hop is facilitated by an ecosystem of OHDSI services through which exemplary ETLs are produced, one for each staging database. The exemplary ETLs are implemented using Pentaho Data Integration (formerly Kettle) (32). Pentaho Data Integration (PDI) is both point and click and self-documenting. PDI is artificially intelligent and uses parallel processing to perform transformations with large databases. During implementation the INSPIRE PDI makes use of a human and machine-readable implementation guide which captures much of the thinking used in the construction of the exemplary ETL.

In between the two hops there are “pass through acceptance criteria”, “data acceptance metrics” and lots of capacity development.

#### Integration with place-based attributes

**Figure 2:**
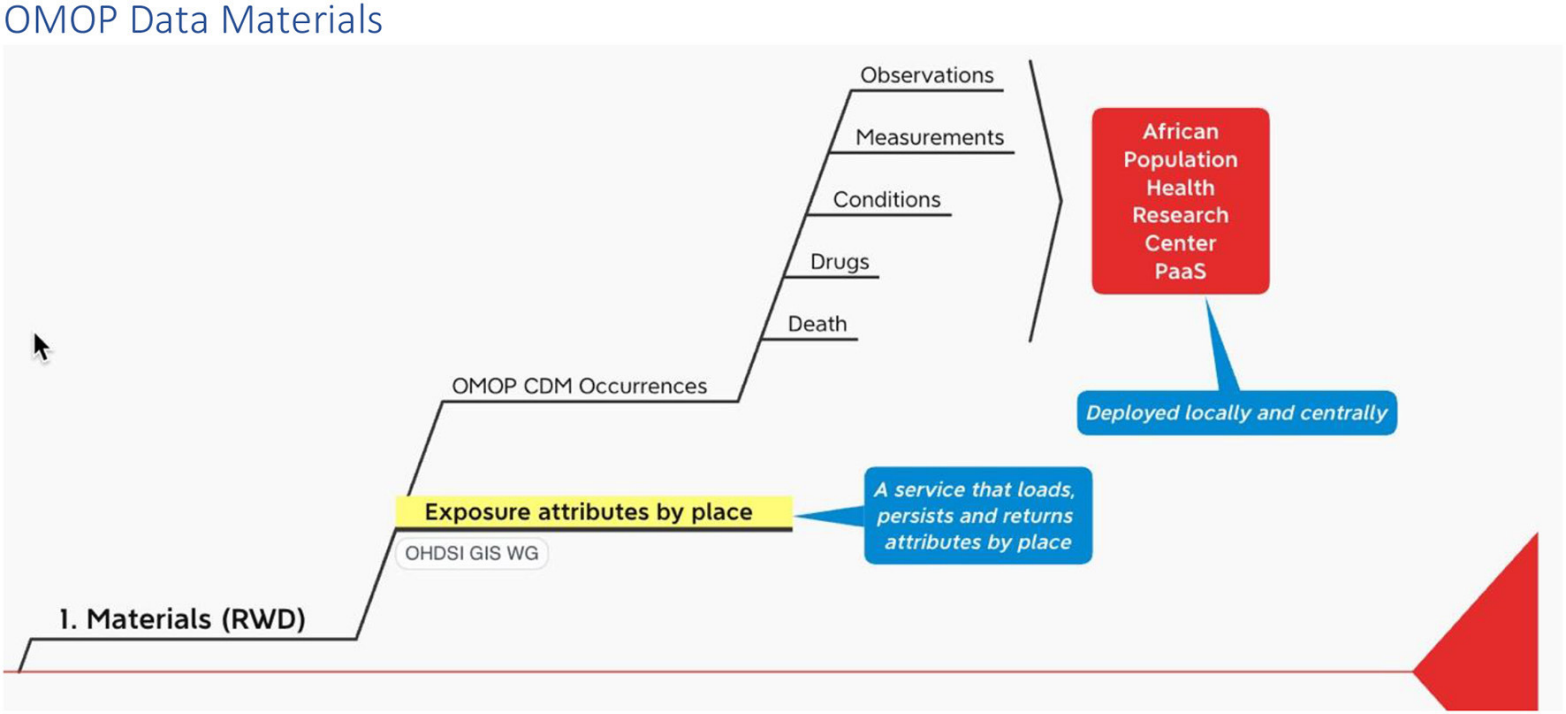
Materials infograph fragment.

Recall that among the materials that INSPIRE is working to include in its PaaS is a service being developed by the OHDSI GIS Working Group that ingests, persists and outputs place-based attributes (Figure 2).

**Figure 3:**
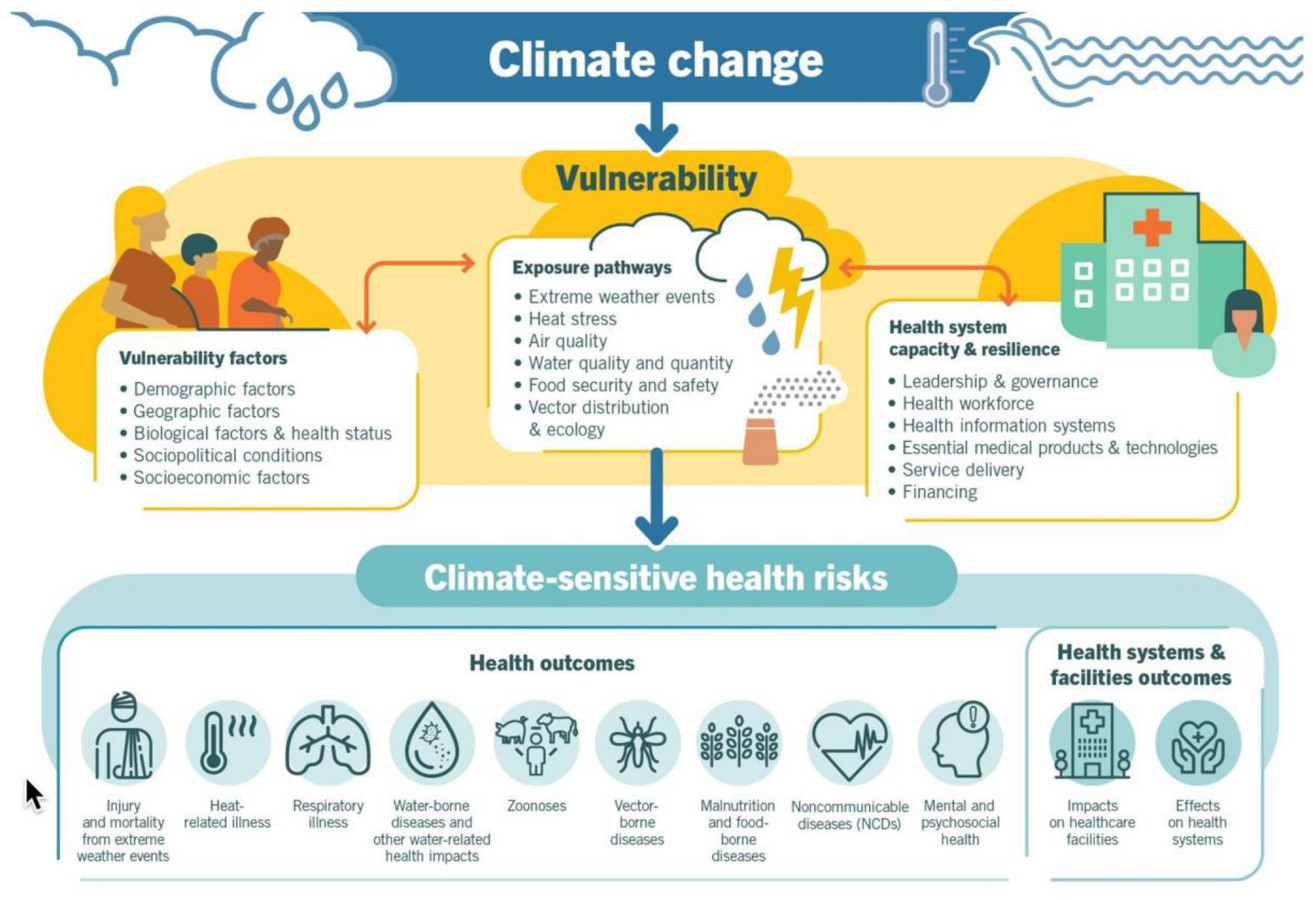
Climate change conditions health (WHO).

According to the World Health Organization (WHO), we need to include support for place-based attributes as a part of our research platform because climate change is the single biggest health threat facing humanity, and health professionals worldwide are already responding to the health harms caused by this unfolding crisis. “More specifically”, climate change is already impacting health in a myriad of ways, including by leading to death and illness from increasingly frequent extreme weather events, such as heatwaves, storms and floods, the disruption of food systems, increases in zoonoses and food-, water- and vector-borne diseases, and mental health issues. Furthermore, climate change is undermining many of the social determinants for good health, such as livelihoods, equality and access to health care and social support structures. These climate-sensitive health risks are disproportionately felt by the most vulnerable and disadvantaged, including women, children, ethnic minorities, poor communities, migrants or displaced persons, older populations, and those with underlying health conditions” (33). The WHO provides this overview of climate-sensitive health risks, their exposure pathways and vulnerability factors, see Figure 3.

This being said, the integration of place-based attributes with the person-level disease and clinical surveillance events we chronicle on our OHDSI platform presents several challenges. These include:

- Defining a best practices we can follow that map the environment to the comings and goings of individuals over time
- Determining a standard vocabulary we might adopt that we can use to label and discriminate among these person-level exposure events for the sake of understanding and analysis

**Figure 4:**
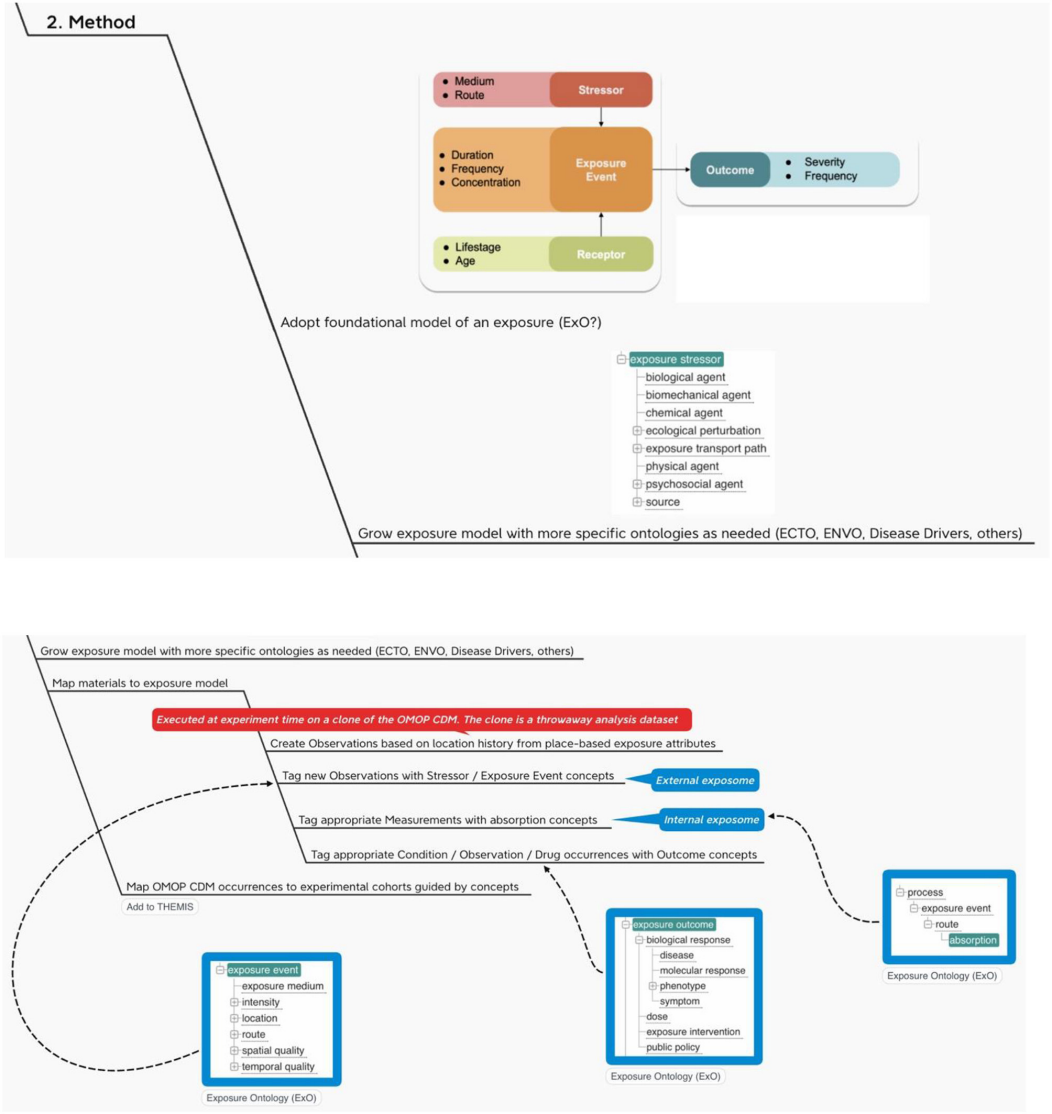
OHDSI adds the exposome.

It is with these challenges in mind that we have developed the following method (Figure 4).

The “four methods” helped create the following infographic which comprises methods, materials and experiments.

## The infographic

**Figure 5:**
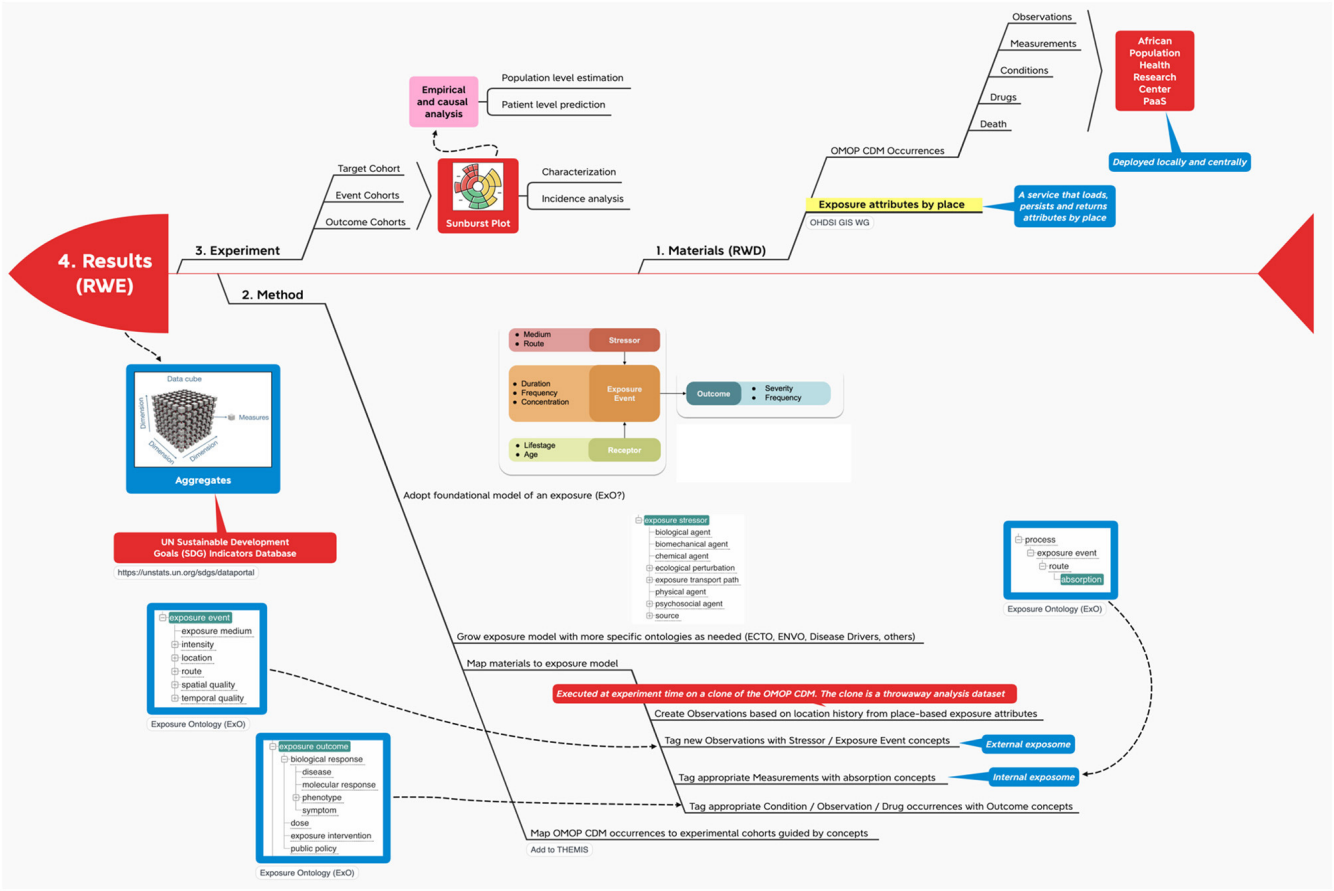
Materials and methods InfographOMOP data materials.

The INSPIRE platform is based on the data sharing principles from N3C, OMOP, OHDSI and the OHDSI place-based extensions to supports research. This section gives an overview of OMOP as a whole in an infograph (see Figure 5). It shows how it can be applied to African population health data, which can include place-based data for climate and other exposomes.

With this method we propose (1) a way to map outputs from the OHDSI GIS Working Group place-based attribute service to OMOP and (2) a standard vocabulary we might use to label the exposure events that accrue to an individual during their comings and goings over time.

The recommendations here are first to create person-level exposure events in the form of OMOP CDM OBSERVATIONs or, alternatively, as exposures in a proposed CDM EXPOSUREs table, depending on the location history of the individual and then to use a foundational model for exposures like Exposure Ontology (ExO) to label these observations (34).

Note that a foundational model for exposures like ExO thinks causally about the environment (stressors) and its effects on individuals (exposure events, receptors and process). In terms of Gartner's Analytic Ascendancy Model (35), the adoption of this type of ontology positions INSPIRE to support not just predictive analytics but also prescriptive analytics. Recall that predictive analytics is more opportunistic when it comes to the selection of predictors. It is bent on predicting what is likely to happen regardless of any causal relations among the predictors. Prescriptive analysis, on the other hand, pays more attention to structure and lends itself to complex decision-making (36).

## Real World Evidence from Real World Data

**Figure 6:**
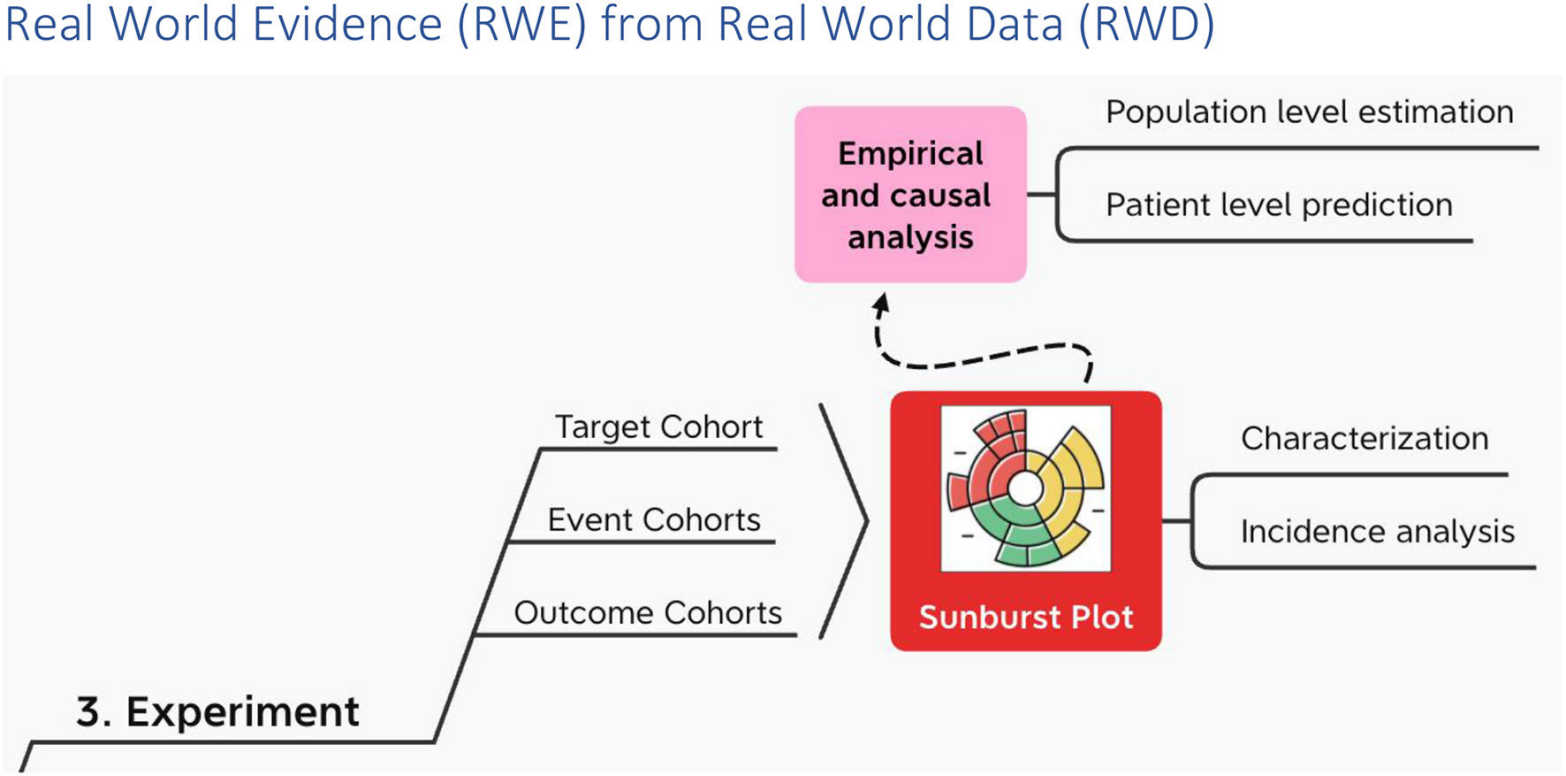
RWE from RWD using OHDSI.

In the experiment the target cohort experiences one or more events in succession or at the same time, leading to various outcomes (Figure 6). Along the way the target cohort at the center of the sunburst plot^2^ is divided again and again by what happens first and then what happens next at each successive division ad infinitum (38).

Each experiment may make use of de novo cohort definitions in OHDSI and/or, alternatively “borrow” cohort definitions from the OHDSI Cohort Library (39).

On top of characterization and incidence analysis (40), i.e., the sunburst plot; using these cohorts, OHDSI performs empirical and causal analysis in the form of patient level prediction (41) and population level estimation (42) respectively. OHDSI packages all of this in a data analysis workbench called ATLAS. And ATLAS provides a user interface through which users can specify and execute these three types of data analysis – descriptive (characterization), predictive and prescriptive (population health) – on top of one or more OMOP CDM databases hosted locally and/or centrally.

Note that predictive analysis uses supervised learning. A target cohort is defined. From the target an outcome cohort is selected based on one or more CDM occurrences (e.g., measurements and/or diagnoses and/or death). With these CDM outcome occurrences, their concepts serve as the labels. Next OHDSI tries to account for these labeled outcomes with predictors that are also extracted from the same target cohort. In OHDSI predictors automatically include demographics as well as perhaps specific occurrences the Principal Investigator (PI) chooses from the target cohort person/patient record. A PI also specifies one or more supervised learning algorithms each with their own hyper-parameter settings (e.g., regularized logistic regression, gradient boosting machines, random forest, K-nearest neighbors, Naïve Bayes, etc.). Finally, predictive analysis tries each of these algorithms to determine which one(s) provide the best fit between the predictors (independent variables and the labeled outcomes (dependent variables).

Predictive analysis in OHDSI uses the same interface called ATLAS used in descriptive and causal analysis. Using ATLAS, a supervised learning experiment can be specified and executed codelessly. In the process ATLAS orchestrates a set of R packages that a user can orchestrate directly in the event specialization is needed. For example, as in attention-based learning, there may be a need that is not empirical to weigh certain predictors and outcomes more than others.

ATLAS also supports network studies through the use of a study package it produces that can propagate analysis settings and results from one OMOP CDM to the next across research organizations and countries. That being said, study packages are not self-explanatory and, as such, require additional FAIRification. As a consequence and for the sake of capacity strengthening in SSA, INSPIRE is participating in the development of an OHDSI tool together with EHDEN and GOFAIR based on schema.org and JSON-LD (43). Using this tool, step-by-step instructions are produced that data scientists can use to guide the construction and execution of observational research studies using ATLAS (See example in the Appendix).

## Implications of OMOP structured results and on-ground challenges

**Figure 7:**
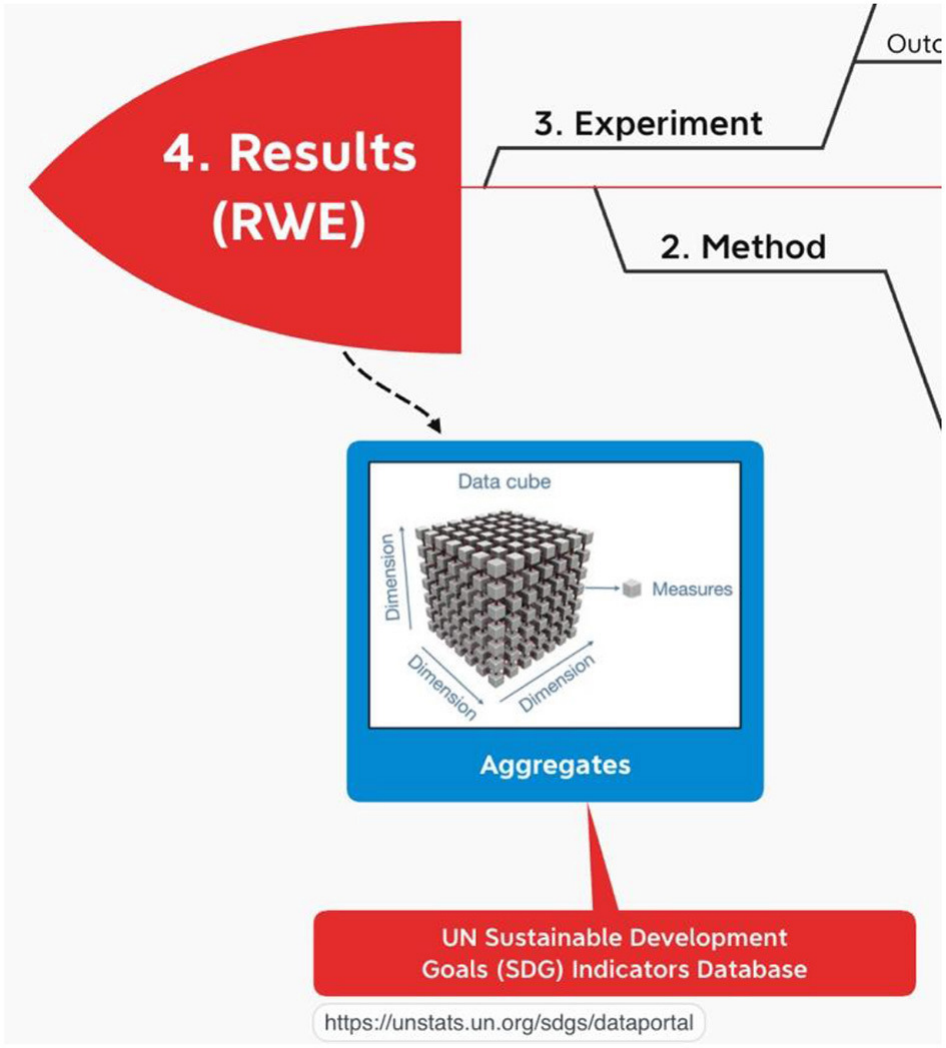
RWE Results (indicators) from using OHDSI.

With federated research, localities report results as aggregates aka “indicators” (Figure 7).

In line with the Global Open Science Cloud (GOSC), INSPIRE, going forward, will be hosting these indicators, disaggregated by sex, age and other factors as applicable and as available, in a datacube. “The datacube crosses different international, national and regional research Infrastructures to create a global digital environment for borderless research and innovation” (11).

INSPIRE is leaning in the direction of SDMX (44) for a datacube implementation of the results from the OMOP CDM. Both the European Commission (45) and the United Nations (46) mandate SDMX for some of their indicator reporting. Guidelines for the Global Data Structure Definition for Sustainable Development Goals Indicators) (47) is the “data structure definition” for the SDMX “dataset” aka “datacube” of sustainable development goals indicators that has been adopted by the UN statistical division. Using SDMX, the OMOP results datacube can report many aggregates by the same sex, location, time period and other factors as applicable and as available. In this way, INSPIRE is able to realize the GOSC goal “to create a *global* digital environment for *borderless* research and *innovation*”.

### Challenges

Whilst the challenges with limited access to data are not unique to COVID-19 disease outbreak, the massive scale of data generated during the outbreak was unique. There were disparate data sources and a lack of integration of data systems in many countries partly due to lack of standardized and harmonized data systems with poor data interoperability. Some vocabularies in the IDSR implementation context were missing in the standard vocabulary mappings and INSPIRE is generating these new vocabularies. We are provisioning location data with aim to account for availability of vaccines and advances in treatment.

Several technical challenges exists in the the implementation context due to limited technical capacity for data management and analysis. Although the ATLAS tool in OHDSI provides a no-code platform for analytics, data managers for both government and private stakeholders require training in the practical application of data science tools to health data issues and to support additional customized solutions. The use of machine learning and AI applications require large quantities of data and computational capacity for training and testing algorithms. Data users often need to upgrade their infrastructure to meet the requirements. There is a pressing need for governments to address affordability issues for internet access. Limited internet connectivity (28%) in Africa coupled with the cost of accessing it with limited infrastructure may exacerbate inequities in public health response.

Furthermore, on the administrative side data comes from multiple sources and and may not be representative of the whole population and issues with duplicates or recording data twice exist. On the cultural spectrum are the biases that exist with legal restrictions on data access and use because of the privacy and confidentiality concerns and only a few countries have legal frameworks for data sharing and use of AI tools. We have developed data sharing agreements with local partners, and limited support for open data sharing of person level data outside the ministry and institutions is still a challenge. INSPIRE is provisioning open source tools with three instances of OHDSI installation for institutions that would like to do own research (i) on a local server or cloud server (ii) a linux installation and (iii) OHDSI on Amazon Web Services to address access and security requirements.

## Discussion

Suffice it to say, when it comes to pandemic preparedness, “It is the platform, stupid!”

Globally the ways in which data are shared are highly variable from raw individual data from participants to aggregates of data published in the public domain. The shared data enable further public health research to inform decisions around pandemic response. Varying standards of data exist including software requirements consequently leading to challenges in data integration and access. Although the ideal situation is that patient level data will be available in real time, most frequently summary data is available. Several efforts have been put in place to support data sharing during COVID-19 and a few studies show that research participants could be advocates for data sharing but there is a lot more variation across regions particularly in SSA. Greater efforts are needed to ensure fairer distribution of benefits accrued. Research shows that unless community engagement in research involve how participants data will be used and who has access, studies risk accentuating health inequalities that exist during a pandemic and underscore those benefits (48).

Despite support to improve data sharing initiatives globally, progress in making individual level data accessible has been slow. Across the African continent, few countries had legal frameworks for sharing data creating challenges both within and between organizations. Data sharing requires data sharing agreements and governance structures for organizations that are involved. Significant improvements are needed in technical and operational knowledge of how data sharing practices and policies can be designed and implemented to be inclusive at both national and international levels in the context of a pandemic response.

Despite these drawbacks, INSPIRE is implementing data sharing framework to facilitate data access and use at national and international levels. Data preparation for a CDM requires significant effort for curating all these data sources and data quality and documentation and may limit the number of institutions who can generate similar research studies.

An evaluation of CDM models shows that OMOP CDM supports best data sharing from longitudinal based EHR studies (49). Although the framework for data curation requires substantial amount of time and resources, the benefits accrued from improved data quality, analytical efficiency with multiple observational data sources and shared OHDSI tools and resources is tremendously useful.

### How this research informs the development of the infographics? How do we measure success?

Indepth discussions of data sharing efforts in many LMICs have largely focused on datasets, adapting a broader view comprising protocols, study materials, lived experience enables a full understanding of the data to enhance replication and address ethical dimensions.

Before the pandemic very few countries have had data sharing frameworks for observational data across domains (diseases, interventions, and the environment). This is true across Africa, Europe and the United States.

Now there are both new initiatives being proposed and facts on the ground. These are in the process of being documented in a landscape analysis by the Global Open Science Cloud (GOSC) Working Group on Sensitive data federation analysis model in population health (50).

Landscape analysis may reveal that, pursuant to the pandemic, when it comes to data sharing frameworks, we are going from dearth to plethora.

In this environment INSPIRE has developed a sustainability model. While in this model INSPIRE is NOT on the bleeding edge, it may as well be for countries that in turn are inspired by its scope and goals. Instead, we aim to be a testbed on a leading edge where significant pathfinder investments are now being awarded. It is this sustainability model that has led INSPIRE to OHDSI away from data catalogs and toward data sharing platforms we can adopt and adapt but, in the final analysis, don't have to build from scratch.

## Conclusion

In sum, the INSPIRE project is a proof of concept through which we can determine whether OHDSI together with a few of its extensions is fit to purpose. Recall that our purpose is to provide decision support when it comes to developing interventions to tame the first in what may be a new generation of pathogens. Like the novel coronavirus SARS-CoV-2, these pathogens may be extremely communicable, quick to morph and hard on our immune systems. The proof of concept is to determine whether *data reuse* and *continuous data analysis* that is the promise of OHDSI can produce the knowledge we need to thwart future pandemics.

This knowledge can be measurement in several ways:

- OHDSI deployments to Health and Demographic Surveillance Systems to build a network for users and producers of longitudinal, population-based health data.
- The establishment of regional OHDSI hubs hosting large numbers of population health and clinical encounters in a federated mixed mode architecture that will produce Findable, Accessible, Interoperable and Reusable (FAIR) data that can be used by researchers and policy makers to answer important policy relevant questions.
- The engagement of MoHs as consumers and producers of OHDSI continuous data analysis with support from the INSPIRE network.
- Publications of cohort studies conducted at the regional OHDSI hubs and policy briefs to improve public health response and evaluate impact on livelihoods.

## Data availability statement

Publicly available datasets were analyzed in this study. This data can be found at: https://www.inspiredata.network.

## Author contributions

SK-M, JT, TB, AT, and JG contributed to conceptualization of the study, design, analysis, interpretation of the data, and writing the manuscript. SK-M and JG wrote the initial draft. All authors contributed to the article and approved the submitted version.

## Footnotes

^1^Note that the same model is being used with EHR data. Each clinic or hospital migrates its own data into a standard format – the WHO COVID-19 Core CRF. Then, an ETL is developed centrally and shared locally that moves the data into each site's OMOP CDM instance.

^2^“The sunburst chart is ideal for displaying hierarchical data. Each level of the hierarchy is represented by one ring or circle with the innermost circle as the top of the hierarchy. A sunburst chart without any hierarchical data (one level of categories), looks similar to a doughnut chart. However, a sunburst chart with multiple levels of categories shows how the outer rings relate to the inner rings” (37).
