# Supplementary material for: Enabling data sharing and utilization for African population health data using OHDSI tools with an OMOP-common data model
Source: Front Public Health. 2023 Jun 9;11:1116682. doi: 10.3389/fpubh.2023.1116682 (PMC10287979; doi:10.3389/fpubh.2023.1116682)

Appendix: Example research in progress using ATLAS

The incidence of diagnosed IDSR priority diseases, conditions and events in 2019, 2020, 2021 and 2222 in SSA disaggregated by country, type of case (OPD, IPD, POE), age, sex, population density (urban/rural) and underlying conditions

**Study Objectives**

- Conduct an [OHDSI ATLAS incidence analysis](https://ohdsi.github.io/TheBookOfOhdsi/Characterization.html#incidence-analysis-in-atlas) of diagnosed IDSR priority diseases, conditions and events in 2019, 2020, 2021 and 2022 across several country-specific OMOP CDM databases (Malawi, Tanzania and Kenya) hosting synthetic data trained from sampled real person-level IDSR data
- Stratify incidence rates in each of the four years by country, type of case (OPD, IPD and POE), age, sex, population density (urban/rural) and underlying conditions reported by the surveillance subject to the health care worker
- Make observations about what happens to IDSR disease, condition and event surveillance in the course of a pandemic
- Produce stratified incidence rates that can be used in what-if simulations

**Target cohorts**

- Persons surveilled in one or more of three country-wide IDSR systems (Malawi, Tanzania and Kenya) in 2019 who were referred to and underwent specimen data collection whose specimen was tested in the lab
- Persons surveilled in one or more of three country-wide IDSR systems (Malawi, Tanzania and Kenya) in 2020 who were referred to and underwent specimen data collection whose specimen was tested in the lab
- Persons surveilled in one or more of three country-wide IDSR systems (Malawi, Tanzania and Kenya) in 2021 who were referred to and underwent specimen data collection whose specimen was tested in the lab
- Persons surveilled in one or more of three country-wide IDSR systems (Malawi, Tanzania and Kenya) in 2022 who were referred to and underwent specimen data collection whose specimen was tested in the lab

**Outcome cohorts**

- One for each IDSR priority disease or condition diagnosed during each of four years (2019, 2020, 2021, 2022)
- If, for instance, ten priority diseases or conditions were diagnosed from specimens collected across the three included IDSR systems in each of the four reporting years, this would result in forty outcome cohorts – one for each condition/year pair.

**Implementation guide**

Study design is captured in a machine-readable implementation guide. The guide instantiates a schema.org MedicalObservationalStudy in a JSON-LD representation. The guide provides assistance to data scientists during the construction and execution of OHDSI research studies using one or more OMOP CDMs and one of the many ATLAS data analytics recipes.


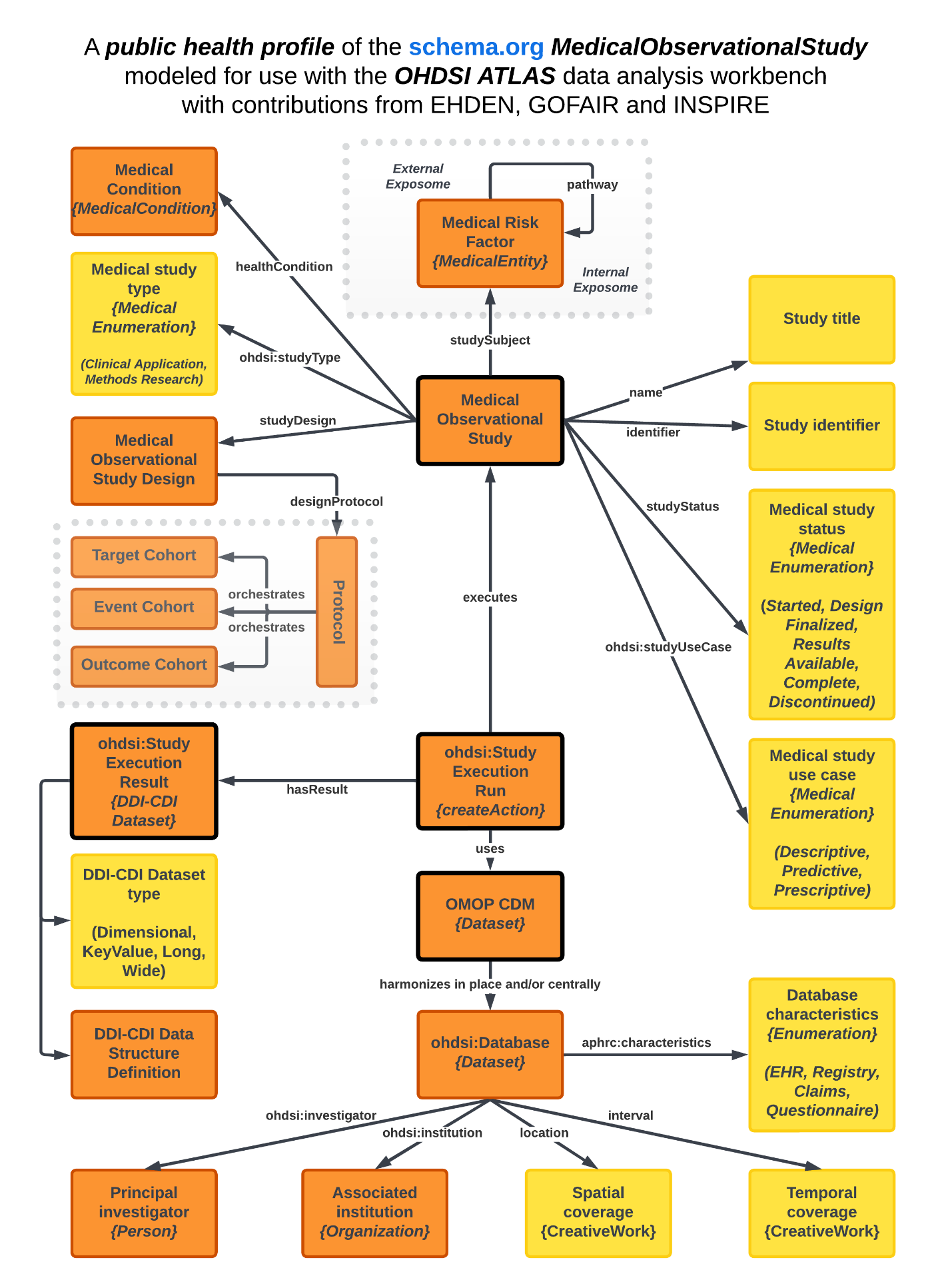

Supplement: Supplementary file 1 [file Data_Sheet_1.docx]
